# A scoping review of high-fidelity bench models for teaching closed reduction of distal radial fractures

**DOI:** 10.15694/mep.2021.000088.1

**Published:** 2021-04-07

**Authors:** Julie Craig, Ian Walsh

**Affiliations:** 1 Royal Victoria Hospital; 2 Queen's University Belfast

**Keywords:** simulation, distal radius, wrist, fracture, closed reduction, reduction, model, high-fidelity, fidelity

## Abstract

This article was migrated. The article was marked as recommended.

Introduction:

Simulation training can be beneficial for developing clinical skills without risks to patients. This review considers the literature on simulation models used for teaching closed reduction (manipulation) procedures for distal radius (wrist) fractures, particularly high-fidelity models, and the evidence supporting the use of such models.

Methods:

A scoping review of Medline and Embase was performed.

Results:

Five articles described low-fidelity models, predominantly focussing on low costs and teaching basic principles. Three articles and two commentary pieces discussing high-fidelity models were identified.

Discussion:

Attitudes towards a high-fidelity simulator were assessed by Egan *et al.* (2013), who found the majority of participants to be in favour of the model’s use as a teaching tool, although participant selection may have been subject to bias.

Mayne *et al.* (2016) subsequently used a high-fidelity model including radio-opaque markers and more objective measurements tools to assess orthopaedic trainees’ closed reduction technique and adequacy of the achieved fracture position and casting. Seniority correlated with higher scores on objective structured assessment of technical skills (OSATS) and global rating scores (GRS) but not radiological measures of fracture position or cast quality, and over 90% of all participants achieved an adequate reduction.

Seeley *et al.* (2017) used radiological measurements and time to task completion with another high-fidelity reduction model. The two most experienced participant groups could not be differentiated on any radiological measures of fracture reduction or on the time taken for reduction, although these groups were significantly better than the most junior participants.

Conclusion:

The discussed models appear helpful to teach inexperienced participants the basic principles and steps in a procedures but a plateau effect appears to limit the potential benefit to more experienced learners. The constraints of educators’ time and financial costs may influence the usage of such models in this type of training.

## Introduction

Simulation training is becoming increasingly adopted in medical education as an adjunct to clinical exposure ( Ruiz-Gómez *et al.,* 2018) as teaching methods focusing on development of competency and patient safety become priorities of education ( Atesok *et al.,* 2012; Kotsis and Chung, 2013). In particular, simulators allow repetitive practice for development of motor skills without risks to patients ( Kneebone, 2003; Papaspyros, Kar, and O’Regan, 2015) in activities such as cast application ( Moktar *et al.,* 2014).

Distal radial fractures are among the most common injuries managed by trauma and orthopaedic departments, occurring in almost 2 cases per 1000 population per year ( Court-Brown and Caesar, 2006). Distal radial fractures are often treated by closed reduction, that is, correction of the fractured bone’s position (reduction) without the use of open surgery. The term manipulation is also commonly used to describe the process of closed reduction of a fracture. Cast application, with appropriate moulding (shaping), is a common method of immobilising the fracture in order to maintain its position.

Failing to achieve or maintain adequate reduction (i.e. an acceptable repositioning of the broken bone) is a common indication for resorting to surgical intervention, although a number of potential complications may be associated with surgery ( Ruedi *et al.,* 2000).

Inadequate reduction, poor cast moulding, and the inexperience of junior trainees performing the procedure all increase the risk of loss of reduction (i.e. deterioration of position) in wrist fractures ( Haddad and Williams, 1995; Mazzini and Martin, 2010).

The technical skills of adequate closed reduction and suitable cast application are therefore extremely important for minimising the risk of requiring surgery, and fracture manipulation (i.e. closed reduction) is recognised as an essential skill for orthopaedic and emergency department trainees by professional bodies in the United Kingdom ( Training Standards Committee of the British Orthopaedic Association, 2015) and United States of America ( Bank *et al.,* 2015; Chapman *et al.,* 2004; Dougherty and Marcus, 2013).

Therefore, simulation training on the technical skill of closed reduction and casting of distal radial fractures could allow inexperienced practitioners to practice this skill without the risk of harm to patients, and could potentially reduce the risk of requiring surgery.

This aim of this review was to assess the literature pertaining to simulation models used for closed reduction simulation training, particularly high-fidelity models of distal radial fracture models, and determine the degree of evidence supporting the usage of such models for education in the closed reduction of distal radial fractures.

## Method

Searches of Medline and Embase using MeSH terms and keyword combinations were performed for English language articles in the field of medical education in humans.

MeSH terms included “simulation training”, “high-fidelity simulation training”, “patient simulation”, “mannequin” (or “manikin” to accommodate American English spelling), in combination with either “closed fracture reduction”, “reduction”, “orthopaedic manipulation”, or keywords commencing “manipulat”. An additional search of keywords commencing in “fractur”, “educat” and “simulat” was also included.

Articles which considered only virtual reality simulation were excluded, but articles which compared a bench model to a virtual reality model were included.

## Results

**Figure 1:**
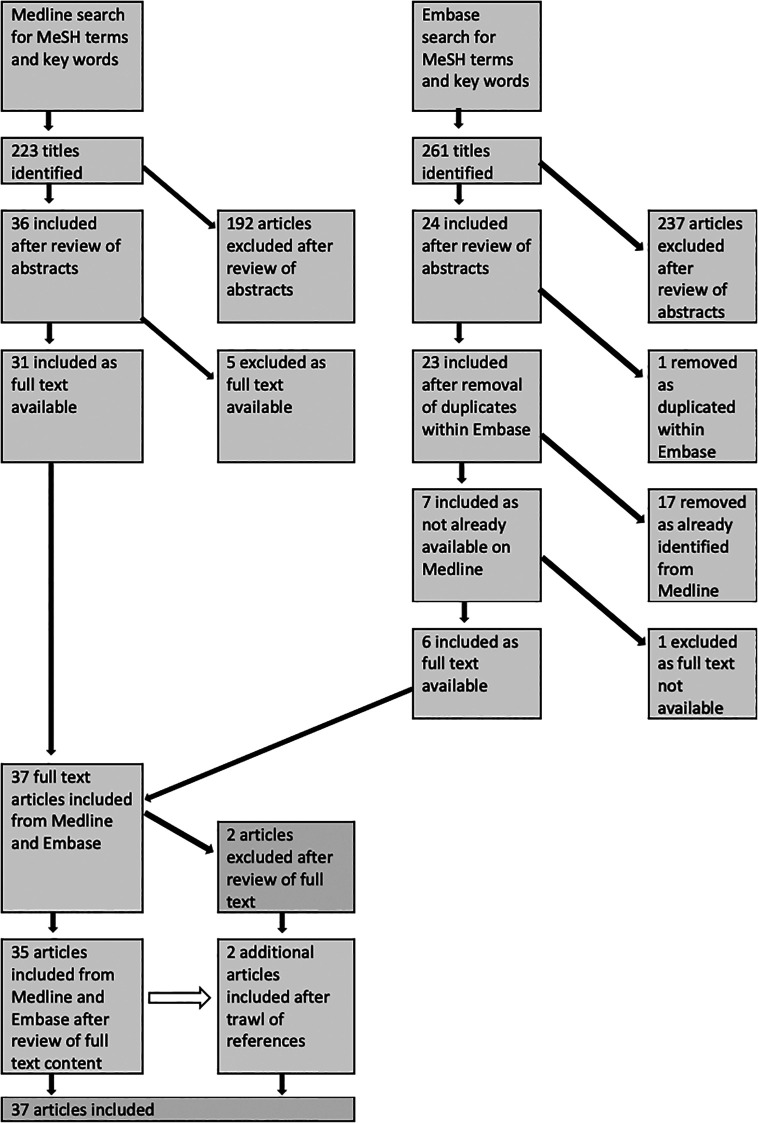
Flow diagram of literature selection process

Searches of Medline and Embase generated 223 and 261 titles respectively. After review of abstracts, removal of duplicates, and exclusion of articles without available full text versions, 37 full text articles were reviewed. Two of these were then excluded as they were purely based on virtual reality simulation training ( Chao *et al.,* 2007; Sugand *et al.,* 2015). Two additional articles were also included after identification from a trawl of the references within the other included articles (Barry Issenberg *et al.,* 2005; Ho, Chimutengwende-Gordon, and Hardy, 2006). The literature selection process is demonstrated in Figure 1.

On reviewing the literature generated from the database searches, it was evident that several key themes were addressed, as shown in Appendix 1.

Five studies used low-fidelity models to explain the principles of fracture reduction ( Berberich *et al.,* 2008; Cox *et al.,* 2016; Ho, Chimutengwende-Gordon and Hardy, 2006; Lopez *et al.,* 2015; Washington *et al.,* 2014). These generally focused on the ease of production rather than demonstrating robust analysis into their effectiveness as educational tools( Cox *et al.,* 2016; Ho, Chimutengwende-Gordon and Hardy, 2006; Washington *et al.,* 2014) and tended to use inexpensive and easily-accessible materials. These models were generally used to explain the basic principles of reduction (i.e. the biomechanics of how a broken bone can be moved), often in a setting where either repetition or inexpensive materials were prioritised, including one study based in a developing country. One exception, ( Berberich *et al.,* 2008) used a novel system of lasers to calculate angles used in cast wedging procedures, but cast wedging is generally not used as part of an initial reduction procedures but as a secondary step if the initial reduction was itself inadequate.

Three further studies simulated other skills associated with distal radius fracture management without fracture reduction, such as cast application or removal ( Bae *et al.,* 2017; Brubacher *et al.,* 2016; Moktar *et al.,* 2014).

Only three articles focused on the use of high-fidelity models for teaching closed reduction of distal radial fractures ( Egan *et al.,* 2013; Mayne *et al.,* 2016; Seeley *et al.,* 2017). Two further commentary pieces ( Kelly and Nolan, 2016; Levin, 2017) referred specifically to the findings of these three articles. The low number of studies in this field was surprising, based on the high incidence of distal radial fractures and the apparent suitability of this injury for simulation training.

In 2013, Egan *et al.* published a study exploring orthopaedic surgeons’ and trainees’ perceptions of a high-fidelity model for teaching closed reduction of distal radial fractures, hypothesising that this would be affected by participants’ level of experience and prior use of simulation training models ( Egan *et al.,* 2013). Egan *et al.* developed a model forearm which included a synthetic bone cut so as to present a simple distal radius fracture pattern. Eccentric tension straps were included to pull the bone out of the normal position if not being manually held in place during the reduction technique. A high-fidelity synthetic soft tissue and skin layer was intended to provide the appearance of a human arm with appropriate soft tissue coverage. A zipper on the underside of the forearm was used to open the simulated arm and allow direct viewing of the position of the bone and the adequacy of the reduction achieved.

Participant recruitment was conducted by an novel method. The authors describe that the simulator was displayed on a table at an international (European) trauma and orthopaedic conference whereby participants were all volunteers who approached the table themselves. This therefore holds a heavy risk of bias, particularly in the context of the study aiming to assess attitudes to this simulator trainer, as these participants were self-selecting, representing members of the orthopaedic community who had a potential interest in such a distal radial simulation model.

Participants performed two attempts of reductions using the model then completed a questionnaire.

Importantly, the recommended reduction technique was based on common steps described in a selection of key texts ( Charnley, 1950; McRae and Esser, 2002; Wheeless, Nunley, and Urbaniak, 2019) demonstrating that the recommended reduction technique was both a long-established accepted method for distal radial fracture reduction and in keeping with current practice.

Among fifty-five included participants, almost half were specialist registrars (i.e. senior trainees), and only nice participants were consultants. The participants’ number of previously-performed wrist reductions (median 100, range 7 - 300) and duration of training (median 3 years, range 0.5 - 25 years) reflected the variability in experience and predominance of trainees. The majority (62%) reported previously using a model to learn an orthopaedic skill, but not specifically fracture management, so this may represent experience in fields such as arthroscopy where simulation training is more established ( Tay, Khajuria and Gupte, 2014).

The majority of participants felt that the model looked like a human forearm (78%), looked and felt like it had a deformity (75% and 80% respectively), and 78% felt that the reduction gave a close approximation to real life. Sixty-four percent of participants mostly agreed that the model allowed them to reduce the fracture by using a similar method to that which they would use in real life but around a third of participants provided neutral or negative responses.

Almost 91% of participants agreed/mostly agreed that the model would be useful to teach the basic steps required for the procedure to someone new to orthopaedics. No significant difference existed between opinions of those with or without previous orthopaedic simulation training experience. However, this finding may be of limited value as it does not necessarily relate to simulation training in upper limb fractures and the participant selection may have been biased. The study recognised a number of other limitations in its design, such as the lack of a validated questionnaire available to assess face validity, and the need to open the soft tissue cover with a zipper to visually inspect the fracture reduction.

The use of radiographic markers to permit assessment of the reduction by x-ray was one of the measures introduced by Mayne *et al.* in their study in 2016, which used a combination of outcome measures to demonstrate the validity of a high-fidelity distal radial model ( Mayne *et al.,* 2016).

Mayne *et al. ( Mayne et al., 2016)* aimed to use objective measurement tools to assess junior and senior orthopaedic trainees performing closed reduction and cast application. Anonymised video recordings of participants performing a simulated wrist reduction were reviewed by two blinded orthopaedic surgeons who attributed scorers using objective structured assessment of technical skills (OSATS) and global rating scores (GRS) as previously developed by Moktar *et al.* using a Delphi technique ( Moktar *et al.,* 2014). Face validity was assessed by participants’ questionnaires.

Radio-opaque markers were used for x-ray assessment of the quality of reduction (i.e. the position achieved, as the number of degrees of palmar tilt in comparison to normal anatomical position) and quality of casting (as the ratio of cast anteroposterior diameter to mediolateral diameter, called the three-point index).

Their model was a modified Sawbones forearm model (Sawbones ®, Malmo, Sweden) with a simulated fracture in the synthetic bone, neoprene straps to provide a deforming force, and metal radio-opaque markers of reduction.

Although no consultants were included among the participants, there was a marked difference in the level of experience between the ten participating junior orthopaedic trainees (averaging five closed reduction procedures), and the ten senior orthopaedic trainees (averaging 49 procedures).

Among questionnaire responses, 85% thought that the model was somewhat realistic or very realistic.

Senior residents achieved significantly higher scores on both OSATS and GRS measures than junior residents, with strong correlations between these two measures.

High levels of inter-rater reliability were demonstrated in OSATS and GRS scoring systems and measurement of radiographic data (palmar tilt and three-point index). However, over 90% of all participants achieved an adequate reduction, with no significant difference identified between junior and senior participants’ palmar tilt measurements or three-point index (casting) results. In addition, OSATS demonstrated no correlation with palmar tilt, and a negative correlation with the three-point index.

In 2017, Seeley *et al. ( Seeley et al., 2017)* published a study aiming to validate a high-fidelity model for teaching distal radial fracture reduction and casting by using purely qualitative outcomes, i.e. radiographic measurements. The modified Sawbones model (Sawbones ®, Malmo, Sweden) had a number of similarities to that used by Mayne *et al. ( Mayne et al., 2016).* The model included radio-opaque paint and filaments paint so that the quality of reduction (amount of residual angulation and displacement of the fracture) could be measured as the primary outcome measure. Time to task completion, the number of x-ray images taken, and the cast index (the ratio of the cast’s measurements in sagittal and coronal planes, as a measure of the quality of casting) were also assessed. Participants were permitted a single attempt to perform a reduction of a simulated distal radius fracture and application of a fibreglass cast, and face validity was assessed using a questionnaire based on that used by Egan *et al. ( Egan et al., 2013).*

All 18 of the participants (six junior residents, four senior residents, and eight attending orthopaedic surgeons) were either orthopaedic trainees on paediatric orthopaedic training rotations or had completed paediatric fellowship training. Also, the study was performed in a paediatric hospital, and the senior author (who was also a developer of the model) was a paediatric orthopaedic surgeon. This has particular implications for context, as it would be more likely that their model and training would be aimed at representing paediatric distal radial fractures and there was no mention of author or developer contributions which may have reflected the management of adult and indeed geriatric wrist fractures.

Eighty-three per cent of participants felt that the model looked, felt and moved like a human forearm. All participants felt that the model provided tactile feedback during reduction and “strongly agreed that the model taught the basics steps of fracture reduction and should be implemented leading training”.

Inter-rater reliability was very high, probably at least in part due to the objective nature of the outcome measures, as measurements of time, distance or angulation.

The two most experienced groups of participants (attending surgeons and senior residents) both performed better than the most inexperienced participants (junior residents) in all radiological measures of fracture reduction and in the time required to complete the reduction. However, senior residents and attending orthopaedic surgeons were statistically similar in these measures. Attending surgeons took less time to apply cast than junior residents, but senior residents were not significantly different from the more experienced and less experienced groups. No differences were identified between any of the groups regarding the number of x-ray images taken or the cast index (denoting cast quality).

## Discussion

Mayne *et al. ( Mayne et al., 2016)* cited Charnley’s comment in “The Closed Treatment of Common Fractures” that “it should be the aim of good manipulative surgeon to know that a fracture has been reduced by his/her sense of touch without after dependence on x-ray” ( Charnley, 1950), seemingly to support the value of high fidelity in simulation as a surgeon’s tactile assessment of reduction is vital. However, the inclusion of radio-opaque markers by Mayne *et al.* and Seeley *et al.* demonstrates that in an age of readily-available high-quality imaging, objective radiographic measures are an essential component of assessing reduction ( Mayne *et al.,* 2016; Seeley *et al.,* 2017).

All three of the articles discussing high-fidelity distal radius fracture reduction simulators ( Egan *et al.,* 2013; Mayne *et al.,* 2016; Seeley *et al.,* 2017), and two commentary pieces ( Kelly and Nolan, 2016; Levin, 2017) which discussed the latter two articles, all recognise the potential benefits of the discussed simulation training systems, particularly given the opportunity for learners to receive feedback on a clinical skill. All three studies’ questionnaires demonstrated good levels of face validity, but results of more objective measures were more mixed.

Mayne *et al.* found that “check list” scoring systems correlated strongly with seniority but these scores did not correspond to improved quality of reduction, and the high proportion of even inexperienced participants achieving “successful” reductions suggested a plateau effect whereby even inexperienced participants achieved similar success rates to experts. In addition, Seeley *et al.* were only able to differentiate reduction quality between the two most experienced groups of participants and the most inexperienced group, but found the two most senior groups (attending surgeons and senior residents) to be indistinguishable.

Both Mayne *et al.* and Seeley *et al.* drew conclusions that while their models could play a valuable role in fracture reduction training, the models studied were more likely to be of benefit in teaching less experienced learners the basic steps required in performing a reduction procedure. However, the high rate of successful reduction among inexperienced participants found by Mayne *et al.,* and the lack of differentiation between all groups found by Seeley *et al.,* suggest that the models used were not sufficiently challenging to represent the spectrum of fracture complexity seen in real clinical practice. This is particularly relevant in a trauma specialty, where a vast range of fracture patterns can be sustained within any single region of a bone, based not only on the orientation of the fracture line but also the degree and pattern of bone fragmentation ( Ruedi *et al.,* 2000). Specifically, Seeley *et al.* made no mention of the potential for this fracture simulator (including its fracture pattern and method of usage) to be much more representative of paediatric and adolescent fractures and bone quality than fractures in adults.

The responses to the questionnaire performed by Seeley *et al.* also eluded to the principle that their high fidelity model would be valuable for teaching the basic steps of fracture reduction, which is often juxtaposed with the suggestion that high-level skills, such as the quality of reduction, may be more difficult to teach to more experienced participants ( Kelly and Nolan, 2016; Mayne *et al.,* 2016).

While the potential value of these models in early training, such as in formative assessment, is generally accepted, their limited ability to differentiate experts from trainees suggest that these models are not yet adequate for use in summative assessments. This also links to the supposition that competency is generally regarded as a benchmark to be reached, sometimes at the expense of recognising that competency in a straight-forward cases does not imply the level of expert experience which may be called for in more challenging cases. In this setting, experienced clinicians would be expected to manage complex fractures which junior trainees may not manage as successfully.

The ( Kelly and Nolan, 2016; Levin, 2017) importance of simulation models in general, and the future potential for simulation training in this field were raised by two commentary pieces ( Kelly and Nolan, 2016; Levin, 2017). Levin cited that “the opportunity to learn procedural and operative skills before utilizing these skills on patients will dramatically help ease the ethical tension of training future surgeons while protecting patient autonomy and the primacy of patient welfare” ( Levin, 2017), and therein lies the likely benefit for such bench models, in providing an opportunity for repeatedly practicing a skill without potential risks to patients.

However, both commentaries balanced these aspirational comments with reflections that the cost, durability, and longevity of such wrist fracture models would influence the availability and usage of such models. Both also recognised that while simulation training does involve less patient contact, it still often requires the involvement of experienced clinicians, either for initial training or to provide feedback on task performance. Therefore, the financial costs of such simulation training programmes are not only related to provision of simulation models but also the need for skilled educators contributing significant time to facilitate such schemes ( Nousiainen *et al.,* 2016).

Kelly and Nolan also highlighted the necessity for such a tool to be “validated by comparing performance on the simulated model with the treatment of actual patients and by evaluating performance before and after training” alluding to more generalised concerns that simulation is extremely difficult to link to patient outcomes, and many studies resort to surrogate measures (such as completion of checklists) even though there is a lack of evidence to confirm that the measured outcomes in simulated settings truly represent potential improvements to patient care.

Particularly in measurements of volar tilt, debate still exists around what may be deemed an acceptable reduction, particularly among the elderly population. A recent review of distal radius management in the elderly ( Luokkala *et al.,* 2020) highlighted that younger patients with different requirements may require different thresholds for acceptable reduction, and a substantial degree of dorsal tilt (i.e. failure to restore volar tilt or even a neutral position) is known to be associated with poor outcomes, but without clear evidence to suggest a cut-off for the required degree of volar tilt.

In an era when value for money is expected in many fields of practice, it should be expected that training programs involving simulation can demonstrate that the time provided by educators is used more effectively than in more traditional teaching methods. None of the 37 articles reviewed as part of this scoping review demonstrated a direct comparison between more traditional educational processes (such as lectures or small group teaching) and simulation training systems, which often also include traditional teaching elements. Levin’s recognition that the cost of simulation training will influence its usage relates not only to the equipment involved but also the personnel that are involved. In simple terms, while much has been taught for decades by experienced educators without the assistance of simulation models, the potential for effective learning from using a model without guidance is very limited.

### Limitations of this review

Some relevant articles may have been excluded on the basis of either publication in languages other than English or due to the lack of availability of full text articles. However, on review of the references from the articles which were gleaned from the Medline and Embase searches, only two additional article was deemed necessary for inclusion. Any articles which did appear repeatedly in the references of the articles had already been included as a result of the literature database search processes.

## Conclusion

Levin’s recognition of simulation’s role in teaching medical procedural skills in a safe setting ( Levin, 2017) is key to the value of simulation training. However “simulation is not real life” ( Jain, Schwarzkopf and Scolaro, 2017). Therefore, it must be considered that the infinite variability of patients’ injuries, and the countless other factors affecting individual patients’ care can never be entirely represented in a single method of simulation. As Kneebone described, a strong case may be made for acquiring basic technical skills in an environment where patient safety is not at risk, and ultimately the needs of the learner must be subordinate to the clinical needs of the patient ( Kneebone, 2003). Even among literature on simulation training of closed fracture management in the early stages of training, there is little evidence that patient outcomes are improved, and reports claiming this sort of success often employ other teaching methods or additional time with expert educators. The benefits of the simulation component of training is therefore seldom clear.

The experience of senior clinicians, and the value they add to teaching of more junior trainees is unlikely to ever be eclipsed by a new technological development such as new simulation training options, and the benefit of such models would appear to be the ability to provide a setting for more basic learning experiences in a way which allows good-quality educators to provide guidance and feedback, repeatedly if needed. Simulation training should complement, not be a substitute for, the immeasurable value of clinical experience ( Ruiz-Gómez *et al.,* 2018).

Among more junior trainees there does appear to be evidence that simulation training is beneficial, if only to instil confidence and familiarity with a procedure which can determine whether or not a patient ultimately requires surgery. However, none of the reviewed literature demonstrated adequate validity from qualitative or quantitative measures to show a significant benefit to senior trainees, for whom their development is based on dealing with a broader spectrum of injuries, for which the standardised nature of simulation training is ill-equipped to represent.

Therefore, this field would benefit from additional research to help determine which types of simulation models, and which levels of fidelity, are of most benefit to trainees and surgeons of differing levels of experience, so that institutions can tailor simulation training to the needs of their learners, and make best use of their finite resources (both educators and finances). There is every likelihood that the emphasis on competency and patient safety will lead to increasing use of simulation training in years to come, but educators and their institutions have a responsibility to ensure that this is employed to maximum effect, rather than purely as a mandatory requirement or fashionable trend. However, the increasing prevalence, and vogue, of simulation training is likely to furnish the medical literature with increasing evidence as to how optimal simulation training opportunities can be provided for learners and assessed in an effective way to support learning. While the literature is still in its infancy for high-fidelity simulators for closed reduction of distal radial fractures, initial positive sentiments from learners and the advent of objective measurement tools to assess learners’ success, are the first promising steps towards developing a cost-effective simulation training system with the potential to support safe learning opportunities for this common and important injury.

Much of the literature reviewed for this scoping review demonstrated that programmes with intensive input from experienced educators and fracture reduction simulation models yield favourable results, the amount of benefit attributable to the simulation model as opposed to the contributing educators has not been quantified. In summary, simulation models can replace the “patient” but not the educator, and in an era when access to both is challenging, the value of skilled teachers should not be overlooked in favour of simulation models.

## Take Home Messages

• Closed reduction (manipulation) of distal radius (wrist) fractures is an important clinical skill.
• Simulation models (both low-fidelity and high-fidelity) are described in the medical literature).
• Three articles describing high-fidelity simulation models all reported participants being in favour of using such models for teaching steps of closed reduction.
• Measures assessing the completion of steps of this clinics skill correlated with seniority, but other more objective measurements (e.g. radiological evidence of reduction and casting quality) demonstrated less consistent findings.
• Such models appear helpful to teach inexperienced participants the basic principles and steps in this procedures but a plateau effect appears to limit the potential benefit to more experienced learners.

## Notes On Contributors

Mrs (Dr) Julie Craig Mb BCh BAO (Hons.) MSc (ortho. eng.) MSc (Clin. Ed). MRCS: Orthopaedic specialty doctor; educational lead for fractures in the Belfast Health and Social Care Trust, Queen’s University lead for final year fracture education.

Mr (Dr) Ian Walsh MD MSc FRCSUrol SFHEA: Consultant Urological Surgeon; Postgraduate Tutor at the Northern Ireland Medical and Dental Training Agency; MSc (with Distinction) in Medical Education; Clinical Academic Teaching Fellow, Deputy Lead for Final Year, and lead for Medical Humanities, Arts in Healthcare at Queen’s University Belfast.

## Figures and Tables

**Appendix 1:** Key themes in the reviewed literature.

Theme	Number of articles (and subcategory if applicable)	Articles
General principles of simulation training and commentary articles	8 articles	Albanese, M., Mejicano, G. and Gruppen, L. (2008) Perspective: Competency-based medical education: A defense against the four horsemen of the medical education apocalypse. *Academic Medicine, 83*(12), 1132-1139. Barry Issenberg, S., McGaghie, W. C., Petrusa, E. R., Lee Gordon, D., *et al.* (2005) ‘Features and uses of high-fidelity medical simulations that lead to effective learning: A BEME systematic review’, *Medical Teacher, 27*(1), pp. 10-28. Kelly, D. and Nolan, B. M. (2016) ‘Models and simulation: The future of orthopaedic residency education?’, *Journal of Bone and Joint Surgery, 98*(5), pp. e21. Kneebone, R. (2003) ‘Simulation in surgical training: Educational issues and practical implications’, *Medical Education, 37*(3), pp. 267-277. Levin, P. E. (2017) ‘CORR Insights®: Teaching the basics: Development and validation of a distal radius reduction and casting model’, *Clinical Orthopaedics and Related Research®, 475*(9), pp. 2306-2307. Mediouni, M. and Volosnikov, A. (2015) ‘The trends and challenges in orthopaedic simulation’, *Journal of Orthopaedics, 12*(4), pp. 253-259. Papaspyros, S. C., Kar, A. and O’Regan, D. J. (2015) ‘Surgical ergonomics - analysis of technical skills, simulation models and assessment methods. *Internal Journal of Surgery, 18, * pp. 83-87. Ruiz-Gómez, J. L., Martín-Parra, J. I., González-Noriega, M., Redondo-Figuero, C. G. *et al.*(2018) ‘Simulation as a surgical teaching model’, *Cirugía Española (English Edition), 96*(1), pp. 12-17.
Models for open reduction and internal fixation of fractures	7 articles	Abagge, M., Uliana, C. S., Fischer, S. T. and Kojima, K. E. (2017) ‘Effectiveness of a step-by-step oral recount before a practical simulation of fracture fixation’, *Injury, 48, * pp. S50-S53. Burns, G. T., King, B. W., Holmes, J. R. and Irwin, T. A. (2017) ‘Evaluating internal fixation skills using surgical simulation’, *Journal of Bone and Joint Surgery, 99*(5), pp. e21. Egol, K. A., Phillips, D., Vongbandith, T., Szyld, D., *et al.* (2015) ‘Do orthopaedic fracture skills courses improve resident performance?’, *Injury, 46*(4), pp. 547-551. LeBlanc, J., Hutchison, C., Hu, Y. and Donnon, T. (2013a) ‘A comparison of orthopaedic resident performance on surgical fixation of an ulnar fracture using virtual reality and synthetic models’ *Journal of Bone and Joint Surgery, 95*(9), pp. e60. LeBlanc, J., Hutchison, C., Hu, Y. and Donnon, T. (2013b) ‘Feasibility and fidelity of practising surgical fixation on a virtual ulna bone’, *Canadian Journal of Surgery, 56*(4), pp. e91. Ruder, J., Turvey, B., Hsu, J. R. and Scanndell, B. P. (2016) ‘Effectiveness of a low-cost drilling module in orthopaedic surgical simulation’, *Journal of Surgical Education, 74*(3), pp. 471-476. Yehyawi, T. M., Thomas, T. P., Ohrt, G. T., Marsh, J. L., *et al.* (2013) ‘ *A simulation trainer for complex articular fracture surgery*’, Journal of Bone and Joint Surgery. *American volume, 95*(13), pp. e92.
Low-fidelity models for teaching the principles of closed reduction of fractures	5 articles	Berberich, T., Reimann, P., Steinacher, M., Erb, T. O., *et al.* (2008) ‘Evaluation of cast wedging in a forearm fracture model’, *Clinical Biomechanics, 23*(7), pp. 895-899. Cox, K., Nilson, J. and Broder, J. S. (2016) ‘An inexpensive, rapidly constructed, durable, and realistic model for orthopedic fracture reduction: 42’, *Academic Emergency Medicine, 23, * pp. S291. Ho, K., Chimutengwende-Gordon, M. and Hardy, J. (2006) ‘A simple model to demonstrate the method of reduction and immobilisation of a forearm fracture in an adult or child’, *The Annals of the Royal College of Surgeons of England, 88*(2), pp. 224-225. Lopez, G., Wright, R., Martin, D., Jung, J. *et al.* (2015) ‘A cost-effective junior resident training and assessment simulator for orthopaedic surgical skills via fundamentals of orthopaedic surgery: AAOS exhibit selection’, *Journal of Bone and Joint Surgery, 97*(8), pp. 659-666. Washington, C. H., Tyler, F. J., Davis, J., Shapiro, D. R., *et al.* (2014) ‘Trauma training course: Innovative teaching models and methods for training health workers in active conflict zones of eastern Myanmar’, *International Journal of Emergency Medicine, 7*(1), pp. 46.
High-fidelity models for teaching cast application or removal without fracture reduction	3 articles	Bae, D. S., Lynch, H., Jamieson, K., Yu-Moe, C. W., *et al.* (2017) ‘Improved safety and cost savings from reductions in cast-saw burns after simulation-based education for orthopaedic surgery residents’, *Joutnal of Bone and Joint Surgery’, 99*(17), pp. e94. Brubacher, J. W., Karg, J., Weinstock, P. and Bae, D. S. (2016) ‘A novel cast removal training simulation to improve patient safety’, *Journal of Surgical Education, 73*(1), pp. 7-11. Moktar, J., Popkin, C. A., Howard, A. and Murnaghan, M. L. (2014) ‘ *Development of a cast application simulator and evaluation of objective measures of performance*’, *Journal of Bone and Joint Surgery, 96*(9), pp.e76.
High-fidelity models for teaching closed reduction of fractures	3 articles	Egan, C., Egan, R., Curran, P., Bryan, K., *et al.* (2013) ‘Development of a model for teaching manipulation of a distal radial fracture’, *Journal of Bone and Joint Surgery, 95*(5), pp. 433-438. Mayne, I. P., Brydges, R., Moktar, J. and Murnaghan, M. L. (2016) ‘Development and assessment of a distal radial fracture model as a clinical teaching tool’, *Journal of Bone and Joint Surgery, 98*(5), pp. 410-416. Seeley, M., Fabricant, P. and Lawrence, J. (2017) ‘Teaching the basics: Development and validation of a distal radius reduction and casting model’, *Clinical Orthopaedics and Related Research ®, 475*(9), pp. 2298-2305.
Other topics	Maxillofacial surgery: 3 articles	Christophel, J. J., Park, S. S., Nogan, S. J. and Essig, G. F. (2017) ‘A facial trauma simulation course for evaluation and treatment of facial fractures’, *JAMA Facial Plastic Surgery, 19*(6), pp. 464-467. D’souza, N., Mainprize, J., Edwards, G., Binhammer, P., *et al.* (2015) ‘Teaching facial fracture repair: A novel method of surgical skills training using three-dimensional biomodels’, *Plastic Surgery, 23*(2), pp. 81-86. Schvartzman, S. C., Silva, R., Salisbury, K., Gaudilliere, D. *et al.* (2014) ‘Computer-aided trauma simulation system with haptic feedback is easy and fast for oral-maxillofacial surgeons to learn and use’, *Journal of Oral and Maxillofacial Surgery, 72*(10), pp. 1984-1993.
	Closed reduction procedures with internal fixation: 1 article	Butler, B. A., Lawton, C. D., Burgess, J., Balderama, E. S., *et al.* (2017) ‘Simulation-based educational module improves intern and medical student performance of closed reduction and percutaneous pinning of pediatric supracondylar humeral fractures’, *Journal of Bone and Joint Surgery, 99*(23), pp. e128.
	Simulation training other than fracture management: 2 articles	Gordon, J. A. and Pawlowski, J. (2002) ‘Education on-demand: The development of a simulator-based medical education service’, *Academic Medicine: Journal of the Association of American Medical Colleges, 77*(7), pp. 751-752. Grant, D. J. and Marriage, S. C. (2012) ‘Training using medical simulation’, *Archives of Disease in Childhood, 97*(3), pp. 255-259.
	Computer-based simulation: 3 articles	Froelich, J. M., Milbrandt, J. C., Novicoff, W. M., Saleh, K. J., *et al.* (2011). ‘Surgical simulators and hip fractures: A role in residency training?’, *Journal of Surgical Education, 68*(4), pp. 298-302. Putman, M. D., Kinnucan, E., Adams, J. E., Van Heest, A. E., *et al.* (2015) ‘On orthopedic surgical skill prediction-The limited value of traditional testing’, *Journal of Surgical Education, 72*(3), pp. 458-470. Rambani, R., Viant, W., Ward, J. and Mohsen, A. (2013) ‘Computer-assisted orthopedic training system for fracture fixation’, *Journal of Surgical Education, 70*(3), pp. 304-308.
	Videos of simulated procedure for education: 1 article	Mehrpour, S. R., Aghamirsalim, M., Motamedi, S. M. K., Larijani, F. A. *et al.* (2013) ‘A supplemental video teaching tool enhances splinting skills’, *Clinical Orthopaedics and Related Research®, 471*(2), pp. 649-654.
	Video recording of real closed reduction procedures for education: 1 article	Jain, N. S., Schwarzkopf, R. and Scolaro, J. A. (2017) ‘Video review as a tool to improve orthopedic residents' performance of closed manipulative reductions’, *Journal of Surgical Education, 74*(4), pp. 663-667.
