# Supplementary material for: A scoping review of high-fidelity bench models for teaching closed reduction of distal radial fractures
Source: MedEdPublish (2016). 2021 Apr 7;10:88. [Version 1] doi: 10.15694/mep.2021.000088.1 (PMC10939654; doi:10.15694/mep.2021.000088.1)
Supplement: Peer review history

# Reviewer report: Reviewer response for version 1

**DOI:** 10.21956/mep.18966.r27155

**Published:** 2021-08-03

**Authors:** Julie Hunt

**Affiliations:** 1 Lincoln Memorial University

This review has been migrated. The reviewer awarded 4 stars out of 5 This paper describes a review of high-fidelity models for reduction of radial fractures. Specifically, 3 studies are discussed in depth, and the strengths and weaknesses of each study is considered and reported. Overall, the paper is helpful for those in the field with an interest in simulating this procedure.There are a few areas in the paper that could be improved. Specifically, the search returned more papers than are discussed, even if only high-fidelity simulators are included. I would have liked to have seen the 2 commentary papers and their opinions/findings included in the results section along with the 3 high-fidelity research papers. Further, it's unclear why/how the Appendix 1 studies were considered--if at all--in the study. If low-fidelity models for radial fracture reduction and casting were also included, which may be of value, then they also should be in the results and discussion.Finally, I would have liked to have seen the authors' criteria for determining low vs high-fidelity. There is often a continuum of fidelity from low to high, and categorizing them into just 2 groups can be challenging.

Reviewer Expertise:

NA

No decision status is available

# Reviewer report: Reviewer response for version 1

**DOI:** 10.21956/mep.18966.r27156

**Published:** 2021-06-17

**Authors:** Ken Masters

**Affiliations:** 1 Sultan Qaboos University

This review has been migrated. The reviewer awarded 3 stars out of 5 An interesting scoping review of bench models for teaching closed reduction of distal radial fractures. The paper begins by noting the importance of simulation training and the specific need to "allow inexperienced practitioners to practice the required skills without the risk of harm to patients." It is with this background that the scoping review has been conducted. The Discussion is detailed and covers most necessary aspects, including possible researcher/result bias.Overall, a useful read that gives some good insight into the topic. There are, however, areas of concern:• The Abstract’s Methods need to give more detail about the search.• In the article’s Methods, more details are required. These would include the dates of the search, who conducted the searches, verification process, etc. • In addition, the exclusion criteria mentioned appear to be limited, as I would assume that other exclusion criteria were applied (e.g. letters, editorials, literature reviews, etc.). These other exclusion criteria need to be identified. (If these were not excluded, then the paper should state that also, just for clarity).• The paper needs to be clear on the results of the articles found. In the search process, it identifies 37 articles, and the Table in the appendix appears to list all of these. So, that is the data set of results. The text of the Results and the Discussion, however, tends to focus on a smaller subset (with just a passing mention of most of the articles). (The Abstract’s Discussion discusses only the three high-fidelity papers.). The authors cannot have it both ways: either their study includes all levels of models, or it includes high-fidelity models only. If all, then all need to be discussed; if high-fidelity only, then the other 34 must be excluded from the Results data set. Whichever decision is made, the article’s title may have to be adjusted also.• The results need to show a full Table 1 of all the items found (using in-text citation format only), including the date of study, type of study, and the most important results of that study.• The Table in Appendix 1 is actually a crucial part of the data, and should not be relegated to an appendix. It should rather be included in the Results as Table 2 (after Table 1 identified in the point above). In order to make it shorter, however, the full listing of the articles does not need to be displayed, merely the standard in-text citation. • The Conclusion appears to introduce new material which is better suited to the Introduction or the Discussion.Minor:Take-home message, second bullet, the end parenthesis needs to be removed.So, a useful study, but the authors need to be clear on the breadth of their criteria, including all studies that meet those criteria, and then discussing all of those. I look forward to Version 2 of the paper in which these issues are addressed.

Reviewer Expertise:

NA

No decision status is available
